# Detection of COVID-19 by quantitative analysis of carbonyl compounds in exhaled breath

**DOI:** 10.1038/s41598-024-61735-7

**Published:** 2024-06-24

**Authors:** Zhenzhen Xie, James D. Morris, Jianmin Pan, Elizabeth A. Cooke, Saurin R. Sutaria, Dawn Balcom, Subathra Marimuthu, Leslie W. Parrish, Holly Aliesky, Justin J. Huang, Shesh N. Rai, Forest W. Arnold, Jiapeng Huang, Michael H. Nantz, Xiao-An Fu

**Affiliations:** 1 Department of Chemical Engineering, University of Louisville, Louisville, KY USA; 2 Division of Biostatistics and Bioinformatics, Department of Environmental and Public Health Sciences, University of Cincinnati College of Medicine, Cincinnati, OH USA; 3 The Cancer Data Science Center, University of Cincinnati College of Medicine, Cincinnati, OH USA; 4 Biostatistics and Informatics Shared Resource, University of Cincinnati Cancer Center, Cincinnati, OH USA; 5 Department of Anesthesiology and Perioperative Medicine, University of Louisville, Louisville, KY USA; 6 Department of Chemistry, University of Louisville, Louisville, KY USA; 7 Division of Infectious Diseases, Department of Medicine, University of Louisville, Louisville, KY USA; 8 DuPont Manual High School, Louisville, KY USA

**Keywords:** COVID-19 detection, Breath analysis, Carbonyl compounds, UHPLC-MS, Microreactor, Biotechnology, Microbiology, Biomarkers, Diseases

## Abstract

COVID-19 has caused a worldwide pandemic, creating an urgent need for early detection methods. Breath analysis has shown great potential as a non-invasive and rapid means for COVID-19 detection. The objective of this study is to detect patients infected with SARS-CoV-2 and even the possibility to screen between different SARS-CoV-2 variants by analysis of carbonyl compounds in breath. Carbonyl compounds in exhaled breath are metabolites related to inflammation and oxidative stress induced by diseases. This study included a cohort of COVID-19 positive and negative subjects confirmed by reverse transcription polymerase chain reaction between March and December 2021. Carbonyl compounds in exhaled breath were captured using a microfabricated silicon microreactor and analyzed by ultra-high-performance liquid chromatography-mass spectrometry (UHPLC-MS). A total of 321 subjects were enrolled in this study. Of these, 141 (85 males, 60.3%) (mean ± SD age: 52 ± 15 years) were COVID-19 (55 during the alpha wave and 86 during the delta wave) positive and 180 (90 males, 50%) (mean ± SD age: 45 ± 15 years) were negative. Panels of a total of 34 ketones and aldehydes in all breath samples were identified for detection of COVID-19 positive patients. Logistic regression models indicated high accuracy/sensitivity/specificity for alpha wave (98.4%/96.4%/100%), for delta wave (88.3%/93.0%/84.6%) and for all COVID-19 positive patients (94.7%/90.1%/98.3%). The results indicate that COVID-19 positive patients can be detected by analysis of carbonyl compounds in exhaled breath. The technology for analysis of carbonyl compounds in exhaled breath has great potential for rapid screening and detection of COVID-19 and for other infectious respiratory diseases in future pandemics.

## Introduction

The World Health Organization (WHO) declared the COVID-19 outbreak caused by SARS-CoV-2 a pandemic in March 2020. The COVID-19 pandemic has had an enormous global economic impact^1^. Subjects infected by SARS-CoV-2 virus show similar symptoms as those of other common respiratory diseases. There were as high as 40% asymptomatic subjects among confirmed COVID-19 patients all over the world^2^. Rapid screening and diagnosis of COVID-19 is a critical tool to curb spreading of the disease. Currently, reverse transcription-polymerase chain reaction (RT-PCR) is considered as the gold standard for screening and diagnosis of COVID-19^3^. Antigen tests have become an important screening tool due to their rapid processing time (15 min) and ease of use at home^4^. However, the nasal/pharyngeal swabs to acquire samples for PCR and antigen tests are uncomfortable and the antigen tests are not able to differentiate SARS-CoV-2 variants^5^.

Breath analysis offers a rapid, and non-invasive detection of diseases and has attracted much attention because of its wide application in medical diagnosis, metabolite bioinformatics and drug discovery^6^. The National Institutes of Health in the United States launched the RADx℠ Radical program to seek innovative, non-traditional diagnostic approaches to address gaps in COVID-19 testing and surveillance (www.radxrad.org) in late 2020. The SCENT program is one of the RADx-rad focus areas for developing new technology platforms to screen for COVID-19 through analysis of volatile organic metabolites in exhaled breath. Volatile organic compounds (VOCs) are generated upon infection by host responses through a series of lipid degradations including ketosis and inflammatory processes present in the lungs^7,8^.

Recently, different methods have been used for analysis of exhaled breath or exhaled breath condensate to detect COVID-19, such as gas chromatography-mass spectrometry (GC-MS)^9,10^, proton transfer reaction time-of-flight mass spectrometry (PTR-ToF-MS)^11–13^, Fourier transform infrared spectroscopy (FTIR)^14^, gas chromatography-ion mobility spectrometry (GC-IMS)^15,16^, sensors and electronic nose^17,18^, and others^19,20^. In April 2022, the US Food and Drug Administration approved the first COVID-19 screening test by GC-MS analysis of breath samples under emergency use authorization (EUA)^21^. Breath analysis techniques also demonstrated the potential for differentiation of variants of SARS-CoV-2^22,23^. Many variants of SARS-CoV-2 create challenges for its detection and curbing the disease. Further, asymptomatic COVID-19 subjects also increase the difficulty to control the spread of infections.

In the present work, we demonstrated a unique approach of using a panel of thirty-four carbonyl compounds detected in all exhaled breath samples for detection of COVID-19, differentiation of Alpha from Delta variant, and detection of asymptomatic COVID-19 infection. Although COVID-19 has faded away because of vaccines, this breath analysis approach could be used for endemic or next pandemic. Carbonyls represent a category of organic molecules from oxidation of lipids^24^ and they play a crucial role in various biological processes, such as inflammation and oxidative stress that strongly occur upon SARS-CoV-2 infection^25^. Carbonyl compounds have been widely detected in breath by both GC-MS and LC-MS^26–28^. We recently developed a microreactor approach coupled with LC-MS for quantitative analysis of a broad range or carbonyl compounds in exhaled breath^28^. Whereas some carbonyl compounds in exhaled breath including acetaldehyde, octanal, acetone and 2-butanone have been reported as biomarkers of COVID-19 in exhaled breath^15,19^, there is no study that focused solely on analysis of carbonyl compounds to diagnose COVID-19. Furthermore, no previous studies have used carbonyl VOCs to differentiate COVID-19 variants or to detect asymptomatic SARS-CoV-2 infection. The objective of this work was to detect SARS-CoV-2 patients by analysis of a broad range of carbonyl compounds in breath and differentiation between the Alpha and Delta variant waves.

## Material and methods

### Study participants

The research protocol of this study was approved by University of Louisville Institutional Review Board (IRB Number 20.1154). All research was performed in accordance with the Declaration of Helsinki and the relevant guidelines/regulations of the IRB. Informed consent was obtained from all participants. Participants were enrolled from the Travel Clinic of the Division of Infectious Diseases at the University of Louisville and the University of Louisville Health Hospitals in Louisville, Kentucky. The Travel Clinic offered COVID-19 PCR testing required prior to international travel, testing for employees of local businesses that required a negative test result prior to returning to their workplace, and for patients requiring a negative PCR test prior to an out-patient surgical procedure. The majority of recruited participants from the Travel Clinic did not exhibit any symptoms of COVID-19 infection and most of the participants were COVID-19 negative from PCR test. Subjects recruited at the hospitals were patients most with mild COVID-19 symptoms and also subjects with trauma and an incidental SARS-CoV-2 positive test. Written informed consent was obtained from each participant. All participants were tested for SARS-CoV-2 using RT-PCR from nasopharyngeal swab samples. Adult patients aged 18 or over were recruited for the study. Both symptomatic and asymptomatic subjects were included. COVID-19 negative subjects were recruited from the Travel Clinic.

### Exhaled breath sample collection and process

A novel silicon microreactor (Fig. S1) was used to capture carbonyl compounds in breath and then the captured compounds were analyzed by ultra high-performance liquid chromatography-mass spectrometry (UHPLC-MS). Exhaled breath samples were collected in 1L Tedlar bags (Sigma-Aldrich, St. Louis, MO) based on our previous study^28,29^. The silicon microreactor was fabricated using microelectromechanical systems (MEMS) technology and the device has been characterized for analyzing carbonyl compounds in exhaled breath^28^. Subjects were instructed to breathe directly into a Tedlar bag through the mouthpiece connected to the bag. A 1 L breath sample of a mixture of tidal and alveolar breath was collected. After collection, the mouthpiece was disconnected, disinfected, and then disposed. The Tedlar bag was sealed with the attached valve and placed in a biohazard bag inside a cooler at 4 °C before transporting to a BioSafety Level 2 Laboratory (BSL-2) for processing and analysis. A nasopharyngeal swab sample for RT-PCR was also collected to test the SARS-CoV-2.

Between March and December 2021, a cohort of subjects with an age range of 18–82 years were recruited for the study. In Louisville, Kentucky, the Alpha variant of SARS-CoV-2 was dominant reported by the City Health Office during the study period between March and June 2021, so subjects recruited during that period of COVID-19 were attributed to the Alpha wave. The Delta variant was dominant between July and December 2021^30^. Thus, subjects recruited during that period of COVID-19 were attributed to the Delta wave.

All breath samples were transferred to the BSL-2 laboratory in the Division of Infectious Diseases Laboratory at the University of Louisville within 2 h of collection for processing. Breath samples were left at ambient temperature for 5 min and then evacuated through the silicon microreactors at a flow rate of 7 mL/min to achieve above 90% capture efficiencies of carbonyl compounds. The silicon microreactor has thousands of triangular micropillars as shown in Fig. S1 (Supporting Information). The fabrication of silicon microreactors is described in a recent publication^28^. The surfaces of the channels and micropillars in the microreactors are functionalized with 2-(aminooxy)ethyl-*N*,*N*,*N*-trimethylammonium triflate (ATM) for capture of aldehydes and ketones via oximation reactions. Tedlar bags were connected to the silicon microreactors through deactivated silica tubes. Breath samples were evacuated from the Tedlar bag through the microreactors, then through HEPA filter, and finally through a 75% alcohol in water impinge before entering into air in a BSL-2 hood to avoid contaminations. Detailed characterization of the silicon microreactors and processing of breath samples were reported elsewhere^28^.

### UHPLC-MS analysis

After the breath sample in the Tedlar bag had been completely evacuated through the microreactors, the ATM reacted adducts were eluted from the microreactor using 200 µL methanol. ATM-acetone-*d*_6_ adduct (5 × 10^–9^ mol) was added as an internal reference (IR) to the eluted samples. Then, the sample was diluted with water by a factor of 10 for analysis. After processing, all materials including tubes and Tedlar bags were decontaminated according to the laboratory standard procedure for biohazardous waste disposal. The samples were analyzed using a Thermo Scientific UHPLC-MS system equipped with an automatic sampler, a Vanquish UHPLC and a Q Exactive Focus Orbitrap Mass Spectrometer (MS). The UHPLC had an ACQUITY BEH phenyl column (2.1 mm × 100 mm, 1.7 μm, Waters, MA, USA) for the separation of ATM-carbonyl adducts. The liquid flow rate through the column was set to 0.2 mL/min. The column temperature was stabilized at 30 °C. The autosampler tray temperature was set at 8 °C. 5 μL of sample volume was injected into the column. The mobile phase A was 0.1% formic acid in water, and mobile phase B was acetonitrile. The mass spectrometer was operated in positive electron spray ionization (ESI) mode with a spray voltage of 3.5 kV. Nitrogen was used as sheath, auxiliary, and sweep gas at flow rates of 49, 12, and 2 (arbitrary units), respectively. Full MS mode with the mass range (m/z) from 50 to 500 with a resolution of 70,000 was used to process the breath samples. For MS/MS analyses, a parallel reaction monitoring (PRM) method was used by MS. Chromatographic separation conditions were set via a gradient elution program^28^. The total chromatographic runtime was 11 min. A total of 34 carbonyl compounds were detected for all breath samples and compound concentrations were calculated by comparison of each compound peak area with that of the IR in each breath sample UHPLC-MS chromatogram, including saturated ketones and aldehydes, hydroxy-aldehydes, unsaturated 2-alkenals, and 4-hydroxy-2-alkenals^28^. A total of 56 features including the 34 carbonyl compound concentrations and 22 derived features of compound ratios and summations including the sum of formaldehyde, acetaldehyde and acetone, the sum of all other carbonyl compounds (OT) and ratios of acetone to butanone were used for statistical analysis (Table S1). Data acquisition and processing were carried out using Thermo Scientific Xcaliber version 4.4. For chemical structure identification of the majority of detected carbonyl compounds, ATM adduct standards were synthesized in-house and used for comparison of retention times and MS/MS spectra^28^.

### Data and statistical analysis

There are many classification methods, which include generalized partial least squares, support vector machines, random forests, and logistic regression model to classify the patients into disease and control groups based on breath analysis data^31^. Prediction (classification) methods involve structured categorical outcome and multiple structured or unstructured covariates^32,33^. There are no models suited for every condition. Therefore, it is important to identify a good model which takes into account sequential structured covariates for the prediction. Furthermore, the proper identification of key carbonyl compounds through statistical and machine learning techniques requires further advances.

In a typical breath sample analysis, the molecular concentration data on several hundred(s) of endogenous and exogenous VOCs are usually obtained. For the detected VOCs, it may not be required to use all VOCs for the patient classification or the predictive model building process (i.e., training the machine learning models and later use them for class label predictions). Therefore, it is pertinent to select/identify a few metabolic VOCs related to COVID-19 as key features for COVID-19 detection. The selection of key features (here metabolic VOCs) out of many VOCs is called feature selection in machine learning^34^. Further, it is essential to determine the number of significant VOCs (e.g., feature size or dimension of VOC data), which can be used in the training of the classification model to predict the class type of COVID-19 patients. The selection of significant VOCs saves time for all VOCs present in the breath samples. Thus, the researchers can focus on a few VOCs instead of generating data on all the VOCs present in breath samples of the patients.

The data was first normalized using logarithm (log_2_) method and then a t-test was used for continuous variables and chi-square test was used for categorical variables^35^. A p-value less than 0.05 defines statistically significant difference at a 95% confidence interval. All calculations were performed with SAS statistical software^36^. A logistic regression model was employed for both univariable and multivariable regressions. After the logarithm and quantile methods to normalize the data, it is no longer non-linear. The multivariable logistic prediction model is the most robust one especially when there are less covariates^32^. The model performance was evaluated by the receiver operator characteristic (ROC) curve with area under the ROC curve (AUC), accuracy, sensitivity, specificity, positive predictive value (PPV) and negative predictive value (NPV). Boxplots were used to visualize the differences between COVID-19 positive and negative groups. A random section of about 67% of samples was used for the training dataset and 33% of samples for testing dataset for all logistic regression models.

## Results

**Figure 1:**
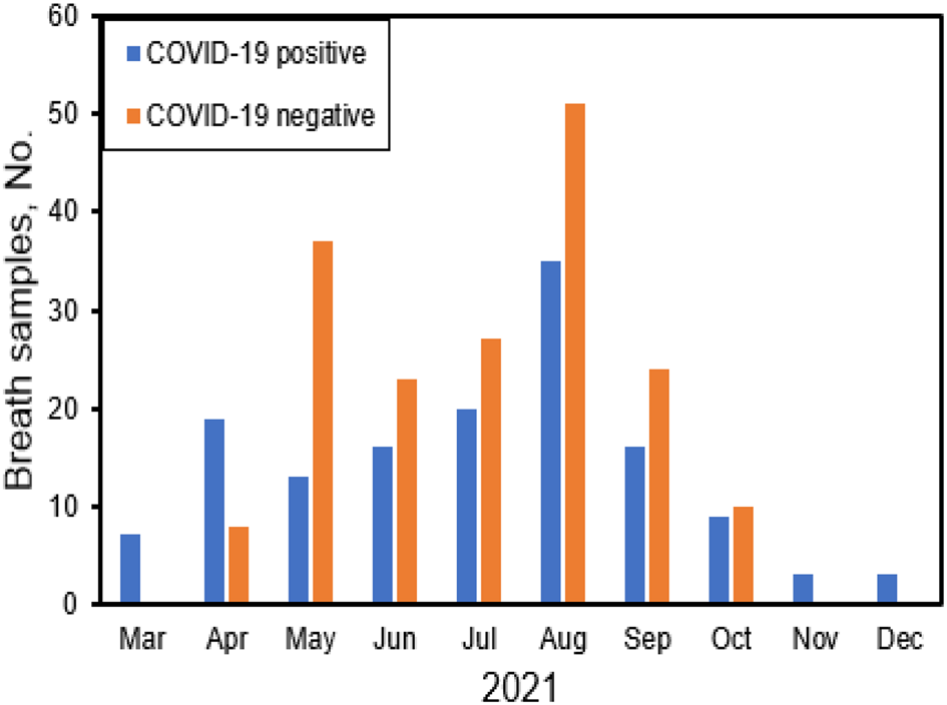
Number of breath samples for COVID-19 positive and negative subjects recruited monthly during the study.

A total of 321 subjects were enrolled. Of these, 141 (85 males, 56 females) were COVID-19 positive and 180 (90 males, 90 females) were negative as confirmed by PCR test. 55 COVID-19 positives were collected during Alpha wave from March to June 2021 and 86 during Delta wave from July to December 2021. Thirty one of the 141 (22%) COVID-19 positive samples were from asymptomatic subjects. The demographic information and the monthly COVID-19 positive and negative participants are presented in Fig. 1 and Table 1.

**Table 1:** Study subject information.

	All subjects	COVID-19 Positive	COVID-19 Negative	p-value (all positives vs all negatives)
Subjects number	321	55 (Alpha), 86 (Delta)	180	–
Vaccinated (n)	156	11 (Alpha), 27 (Delta)	118	–
Age (years) (mean ± SD)	48 ± 15	52 ± 15	45 ± 15	0.0004
Male (n)	175	85	90	0.0934
White race	228	92	136	0.0570
Height (ft) (mean ± SD)	5.66 ± 0.33	5.70 ± 0.32	5.64 ± 0.35	–
Weight (lb) (mean ± SD)	192 ± 51	208 ± 58	179 ± 40	–
BMI (kg/m^2^)	29.2 ± 7.4	31.4 ± 8.9	27.5 ± 5.4	0.0000

### SARS-CoV-2 alpha wave

**Figure 2:**
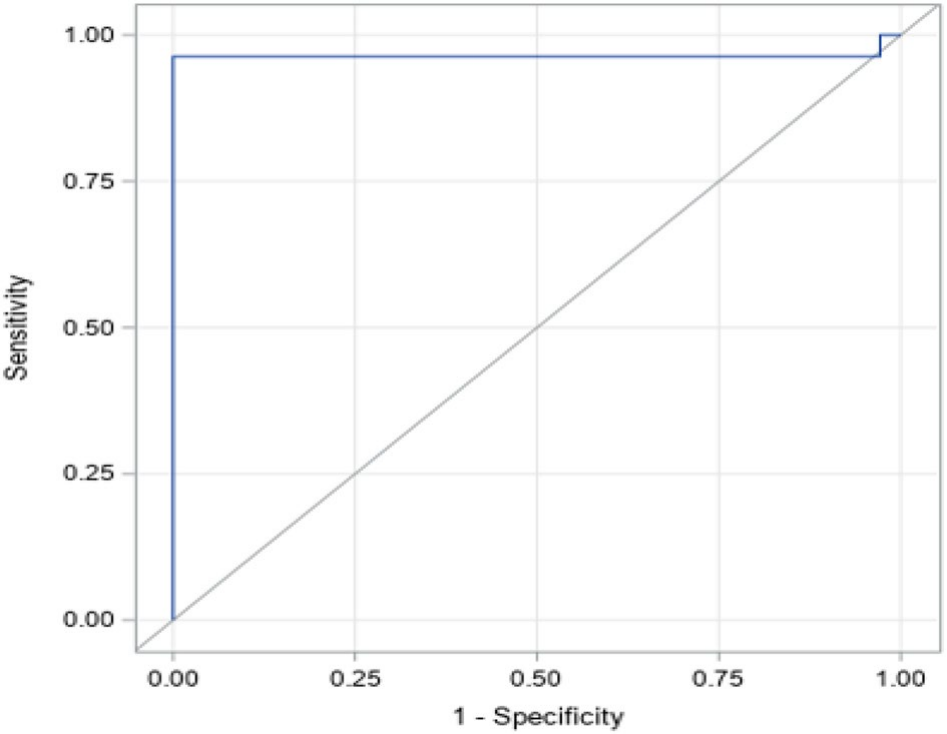
Receiver operator characteristics (ROC) curve (AUC = 0.9647) for multivariable model to distinguish COVID-19 Alpha wave positive and negative groups with two features of acetone/2-butanone ratio and 2-pentenal/OT.

The Alpha variant spread quickly in 2020 in United Kingdom and soon became the dominant variant in the U.S. as well. There were 55 samples positive for the Alpha wave, and 70 negative samples collected from March to June 2021. Twenty two out of the 56 features showed significant differences between positive and negative (Table 2). Table S1 indicates all 56 features, chemical formula, m/z and retention times. The best performing multivariable logistic model was generated using only two features (ID # 41: acetone/2-butanone and 52: 2-pentenal/OT) to distinguish COVID-19 Alpha wave positive group from negative group with a sensitivity of 96.4%, specificity of 100%, PPV of 100%, NPV of 97.2%, an overall accuracy of 98.4% and an AUC of 96.5%. Boxplots of the two features are shown in Fig. S2. ROC curve for the model is shown in Fig. 2. The decrease of 2-pentenal/OT in COVID-19 positive subjects indicates that there was less 2-pentenal in the sum of all other detected carbonyl compounds excluding formaldehyde, acetaldehyde and acetone (Fig. S2). Acetone/2-butanone ratio as a marker of COVID-19 has been reported^15^. Acetone and many aldehydes including butanal, pentanal, hexanal, heptanal, and octanal were elevated in COVID-19 Alpha wave patients (Table 2). These compounds were reported as biomarkers for COVID-19^11,15,19^.

**Table 2:** VOC biomarkers selected to distinguish between different groups.

ID	Features name	Alpha (55) vs Negative (70)	Delta (86) vs Negative (110)	All Positive (141) vs Negative (180)	Asymptomatic (31) vs Negative (183)
1	Formaldehyde		↓		
2*	Acetaldehyde	↑	(P = 0.212)	↑	↑
3*	Acetone	↑	↑	↑	↑
5*	Butanal	↑	(P = 0.156)	↑	↑
7	Pentanal	↑			
8*	Hexanone	↑			↑
9*	Hexanal	↑	↓	↓	(p = 0.466)
10*	Heptanal	↑			↑
11*	Octanal	↑	↓	(p = 0.078)	↑
12	Nonanal				↑
16*	Acrolein		↓	↓	↓
17*	Crotonaldehyde		↓	↓	
18*	2-Pentenal		↓	↓	(p = 0.0578)
19*	2-Nonenal		↓	↓	
22*	Malondialdehyde	↑	↓		
23*	Hydroxy-pentenal		↓	↓	↓
25*	Hydroxy-heptenal	↑		↑	
27	4-HNE				↓
28	Hydroxy-acetaldehyde				↑
29	Hydroxy-acetone	↑			
30	Hydroxy-2-butanone	↑			
31*	Hydroxy-pentanal	↑	↑	↑	(0.116)
32*	Hydroxy-hexanal	↑	↓		↑
34*	4-HHE	↑		↑	
35*	Total of formaldehyde + acetaldehyde + acetone (C1 + C2 + C3)	↑	↑	↑	↑
36*	Other total (excluded C1, C2, C3)	↑	↓		
37*	Formaldehyde/(C1 + C2 + C3)	(p = 0.247)	↓	↓	↓
40*	Acetone/formaldehyde		↑	↑	↑
41*	Acetone/2-butanone	↑	(p = 0.091)	↑	↑
42	2-Butanone + butanal + 2-pentanone + pentanal (C4 + C5)	↑	(p = 0.060)		
45*	Butanal/OT	↑	↑	↑	↑
50	Octanal/OT				↑
51	Nonanal/OT	(p = 0.078)	(p = 0.075)		↑
52*	2-Pentenal/OT	↓		↓	
54*	Hydroxy-acetaldehyde/ OT		↑		↑
56	Hydroxy-2-butanone/OT	↑			

ID, identification.

*Indicates that the VOC appears in multiple biomarker sets.

### SARS-CoV-2 delta wave

There were 86 samples positive for the Delta wave and 110 negative samples from July to December 2021. Eighteen out of 56 features showed significant differences between Delta positive and negative (Table 2). There were only two elevated compounds acetone and hydroxy-pentanal in common for both Alpha and Delta waves. A new set of 12 biomarkers (ID # 9, 17, 22, 23, 31, 32, 35, 36 37, 41, 42, and 45, see Table 2) were used to generate the multivariable logistic model to distinguish COVID-19 Delta wave period positive and negative group with a sensitivity of 93.0%, specificity of 84.6%, PPV of 82.5%, NPV of 93.9%, an overall accuracy of 88.3% and an AUC of 93.3% (Table 3). Figures S3 and S4 show the boxplots of the 12 biomarkers and ROC curve of the model, respectively.

**Table 3:** Summarized accuracy, sensitivity, specificity, PPV and NPV with lower and upper 95% confidence interval for each group.

%	Sensitivity	Specificity	PPV	NPV	Accuracy	Features
Alpha period positive vs negative	96.4 (87.0–99.7)	100 (93.8–100)	100 (91.9–100)	97.2 (89.9–99.8)	98.4 (94.0–99.9)	Acetone/2-butanone ratio, 2-pentenal/OT
Delta period positive vs negative	93.0 (85.3–97.1)	84.6 (76.5–90.2)	82.5 (73.6–88.9)	93.9 (87.2–97.5)	88.3 (83.0–92.1)	Hexanal, crotonaldehyde, malondialdehyde, hydroxy-pentenal, hydroxy-pentanal, hydroxy-hexanal, C1 + C2 + C3, other total (excluded C1, C2, C3), formaldehyde/(C1 + C2 + C3), acetone/2-butanone, C4 + C5, butanal/OT
All positive vs all negative	90.1 (83.9–94.1)	98.3 (95.0–99.7)	97.7 (93.1–99.5)	92.7 (88.0–95.7)	94.7 (91.6–96.7)	Acetone, hexanal, C1 + C2 + C3, acetone/2-butanone and butanal/OT
Alpha positive vs Delta positive	100 (94.9–100)	100 (92.2–100)	100 (94.9–100)	100 (92.2–100)	100 (96.8–100)	Butanal, pentanal, malondialdehyde, octanal/OT
Asymptomatic vs all negative	71.0 (53.2–84.1)	99.5 (96.7–100)	95.7 (77.3–100)	95.3 (91.2–97.6)	95.3 (91.5–97.8)	Acetone, hydroxy-hexanal, butanal/OT, acetone/2-butanone

### All COVID-19 positive and negative subjects

All the 141 COVID positive and 180 negative breath data were analyzed together. Eighteen out of 56 features showed significant differences between all COVID-19 positive group and all COVID-19 negative group with p-value < 0.05. A multivariable logistic model containing a set of five biomarkers (ID # 3: acetone, 9: hexanal, 35: total of formaldehyde and acetaldehyde and acetone, 41: ratio of acetone/2-butanone and 45: ratio of butanal/OT, see Table 2) were able to differentiate COVID-19 positive from negative groups regardless of variants, with a sensitivity of 90.1%, specificity of 98.3%, PPV of 97.7%, NPV of 92.7%, an overall accuracy of 94.7% and an AUC of 93.3%. Figures S5 and S6 show the boxplots of the five and ROC curve of the multivariable logistic regression model, respectively.

### SARS-CoV-2 alpha wave vs delta wave

We also compared Alpha wave with Delta wave breath samples to examine the VOC signature differences between variants. A multivariable logistic model with four biomarkers (malondialdehyde, butylaldehyde, pentanal, ratio of octanal/OT) were able to distinguish Alpha positive and Delta positive with above 90% sensitivity and specificity. The box plots of the four biomarkers and ROC curve are shown in Figs. S7 and S8. These results indicate that the breath signatures vary as different variants emerge, which may impact the future breath analysis on diagnosing COVID-19 and other viral respiratory infections. It was reported that Delta variant caused more severe inflammation than Alpha variant^37^. Delta variant was significantly more transmissible than the Alpha variant^38^. McCartney et al.^23^ and Sharma et al.^22^ reported that both sensitivity and specificity were significantly improved when modeling the Delta wave and the Omicron wave separately.

### Asymptomatic COVID-19 positive vs negative

Asymptomatic SARS-CoV-2 positives were separated and compared with all negatives. Out of 141 COVID-19 positive subjects, 31 (Alpha n = 12, Delta n = 19) were asymptomatic positive subjects. In this analysis, we compared 31 asymptomatic COVID-19 positive subjects with all 180 negative subjects. Twenty out of 56 features show significant differences with p-value < 0.05. A sensitivity of 71.0%, specificity of 99.5%, PPV of 95.7%, NPV of 95.3%, an overall accuracy of 95.3% and an AUC of 88.0% were achieved with a logistic model of four biomarkers (ID # 3: acetone, 32: hydroxyhexanal, 41: acetone/2-butanone, 45: butanal/OT in Table 2). The separated logistic regression models for Alpha and Delta provide much higher sensitivity for asymptomatic SARS-CoV-2 positive subjects as shown above. Boxplots of the four biomarkers and ROC curve of the multivariable logistic model are shown in Figs. S9 and S10. The sample size of asymptomatic COVID-19 positive patients is small, which is a limitation of this comparison.

## Discussion

Three different logistic regression models were developed for differentiation of COVID-19 positive from negative using samples from Alpha, Delta waves and a combination of all Alpha and Delta. Table 3 lists the sensitivities, specificities, NPV, PPV and accuracies with lower and upper 95% confidence interval for each group. The models showed similar or better results than previous breath analysis reports involving COVID-19 patients^9,12,14–16,21–23^. As indicated in Table 3, sensitivity is higher when we modeled the waves separately. The model for prediction of Alpha positives had the highest sensitivity (96.4%), followed by that for Delta (93.0%) and combined Alpha and Delta (90.1%). The specificity, or true negative rate was 100% for Alpha period model, followed by the combined model (98.3%) and Delta period model (84.6%).

The VOCs affected by SARS-CoV-2 in exhaled breath could be from the host response to infection. Inflammation responses could be induced by rapid SARS-CoV-2 viral replication, cellular damage, angiotensin-converting enzyme-2 downregulation, and anti-spike protein-neutralizing antibodies (anti-S-IgG)^25^. The inflammation responses to SARS-CoV-2 cause oxidation of lipids which leads to higher levels of specific carbonyl compounds in the respiratory tracts and lungs^24^. Liangou reported that acetone, acetaldehyde, heptanal, octanal, and 2-butanone were elevated in COVID-19 positive patients^11^. Ruszkiewicz could discriminate COVID-19 from other conditions by ketones (acetone, acetone/2-butanone cluster) and aldehydes (ethanal, propanal, heptanal, octanal)^15^. Berna found octanal, nonanal, and heptanal with elevated concentrations for COVID-19 infected patients^19^. However, there was no virus variant in these studies. This work identified acetone, butanal, pentanal, hexanal, and the acetone/2-butanone ratio as biomarkers for COVID-19.

One limitation of the current work is the lack of cross reactivity validation. Subjects infected by other viruses, such as influenza, respiratory syncytial virus (RSV), adenovirus, rhinovirus and bacterial pneumonia may exhibit similar symptoms as COVID-19 patients. Prior studies demonstrated the metabolite differences in breath from respiratory pathogens other than COVID-19^39,40^. Steppert et al.^20^ and Ruszkiewicz et al.^15^ reported that breath analysis could distinguish COVID-19 and influenza infections in a small cohort study by using MCC-IMS and GC-IMS, respectively. More studies on the VOC signatures by comparing COVID-19 and other respiratory infection may improve the machine learning model for better diagnosis. This breath analysis technology can be easily adopted for detection of other respiratory infection in future endemic and pandemic diseases.

## Conclusion

UHPLC-MS in combination with a MEMS-fabricated silicon microreactor was used to analyze carbonyl compounds in exhaled breath to differentiate COVID-19 positive and negative subjects. The sensitivity increased when modeling variants separately. The overall results are promising for SARS-CoV-2 detection. When all positive (both Alpha and Delta) and all negative samples were used for training a logistic regression algorithm, the model still achieved 90.1% sensitivity, 98.3% specificity and 94.7% accuracy. A study to compare the difference between SARS-CoV-2 and other respiratory pathogens is required to fully verify the method. The method could be adopted for detection of other contagious respiratory viral diseases for curbing future endemic and pandemic diseases.

### Supplementary Information

Supplementary Information.

## Data Availability

Data and data dictionary are available with the publication at https://radx-hub.nih.gov/home.
