# Supplementary material for: Detection of COVID-19 by quantitative analysis of carbonyl compounds in exhaled breath
Source: Sci Rep. 2024 Jun 24;14:14568. doi: 10.1038/s41598-024-61735-7 (PMC11196736; doi:10.1038/s41598-024-61735-7)
Supplement: Supplementary file 1 — Supplementary Information. [file 41598_2024_61735_MOESM1_ESM.docx]

**Detection of COVID-19 by quantitative analysis of carbonyl compounds in exhaled breath**

Zhenzhen Xie^1^ , James D. Morris^1^, Jianmin Pan^2^, Elizabeth A. Cooke^3^, Saurin R. Sutaria^4^, Dawn Balcom^5^, Subathra Marimuthu^5^, Leslie W. Parrish^5^, Holly Aliesky^5^, Justin J. Huang^6^, Shesh N. Rai^2^, Forest W. Arnold^5^, Jiapeng Huang^3*^, Michael H. Nantz^4*^, and Xiao-An Fu^1*^

^1^ Department of Chemical Engineering, University of Louisville, Louisville, KY, USA

^2^ Division of Biostatistics and Bioinformatics, Department of Environmental and Public Health Sciences, University of Cincinnati College of Medicine, OH, USA

^3^  Department of Anesthesiology and Perioperative Medicine, University of Louisville

^4^  Department of Chemistry, University of Louisville

^5^ Division of Infectious Diseases, Department of Medicine, University of Louisville

^6^ DuPont Manual High School, Louisville, Kentucky, USA

***** Correspondence: [xiaoan.fu@louisville.edu](mailto:xiaoan.fu@louisville.edu); Tel:1-502-852-6349; [jiapeng.huang@louisville.edu](mailto:jiapeng.huang@louisville.edu), [michael.nantz@louisville.edu](mailto:Michael.nantz@louisville.edu); Tel: 1-502-852-8069

**Table S1.** All 56 features (Compounds and compound ratios) used for the study

| **#** | **RT (min)** | **Putative identification** | **Formula** | **m/z** | **acc. (ppm)** |
| --- | --- | --- | --- | --- | --- |
| 1 | 1.38 | formaldehyde | CH2O | 131.1177 | 1.144 |
| 2 | 2.06 | acetaldehyde | C2H4O | 145.1333 | 1.516 |
| 3 | 2.99 | acetone | C3H6O | 159.1489 | 1.634 |
| 4 | 3.45 | 2-butanone | C4H8O | 173.1645 | 1.848 |
| 5 | 3.64 | butanal | C4H8O | 173.1645 | 1.848 |
| 6 | 3.94 | 2-pentanone | C5H10O | 187.1802 | 1.71 |
| 7 | 4.23 | pentanal | C5H10O | 187.1802 | 1.71 |
| 8 | 4.47/4.50 | 2-hexanone | C6H12O | 201.1957 | 2.038 |
| 9 | 4.82 | hexanal | C6H12O | 201.1957 | 2.038 |
| 10 | 5.28/5.33 | heptanal | C7H14O | 215.2114 | 1.998 |
| 11 | 5.68/5.76 | octanal | C8H16O | 229.2269 | 2.356 |
| 12 | 6.12/6.21 | nonanal | C9H18O | 243.2426 | 1.932 |
| 13 | 6.96/7.17 | decanal | C10H20O | 257.2582 | 2.216 |
| 14 | 8.25/8.45 | undecanal | C11H22O | 271.274 | 1.585 |
| 15 | 9.15/9.30 | dodecanal | C12H24O | 285.2893 | 2.524 |
| 16 | 3.07 | acrolein | C3H4O | 157.1333 | 1.527 |
| 17 | 3.55/3.63 | crotonaldehyde | C4H6O | 171.1489 | 1.636 |
| 18 | 4.04/4.16 | pentenal | C5H8O | 185.1646 | 1.566 |
| 19 | 4.61/4.76 | hexenal | C6H10O | 199.1802 | 1.607 |
| 20 | 5.53/5.67 | octenal | C8H14O | 227.2111 | 2.905 |
| 21 | 5.96/6.21 | nonenal | C9H16O | 241.2269 | 2.28 |
| 22 | 1.38/2.11 | malondialdehyde | C3H4O2 | 137.1181 | 2.29 |
| 23 | 3.3 | 4-hydroxy-2-pentenal | C5H8O2 | 201.1594 | 1.496 |
| 24 | 3.45 | 4-hydroxy-2-hexenal | C4H6O2 | 215.1749 | 1.988 |
| 25 | 3.75 | 4-hydroxy-2-heptenal | C7H12O2 | 229.1911 | 2.138 |
| 26 | 4.25 | 4-hydroxy-2-octenal | C8H14O2 | 243.2062 | -0.218 |
| 27 | 4.91 | 4-hydroxy-2-nonenal | C9H16O2 | 257.2218 | 2.22 |
| 28 | 1.29/1.36 | hydroxy-acetaldehyde | C2H4O2 | 161.1281 | 1.986 |
| 29 | 1.42 | hydroxy-acetone | C3H6O2 | 175.1438 | 1.77 |
| 30 | 2.16/2.33 | hydroxy-2-butanone | C4H8O2 | 189.1594 | 2.009 |
| 31 | 3.32/3.48 | hydroxy-pentanal | C5H10O2 | 203.1751 | 1.624 |
| 32 | 3.56/3.84 | hydroxy-hexanal | C6H12O2 | 217.1906 | 2.302 |
| 33 | 4.37/4.49 | hydroxy-heptanal | C7H14O2 | 231.2061 | 2.465 |
| 34 | 3.11 | hydroxy-butenal | C4H6O2 | 187.1438 | 1.502 |
| 35 |  | formaldehyde+acetaldehyde+acetone | C1+C2+C3 |  |  |
| 36 |  | All summation excluded C1, C2, C3 | C4+..+C12 (OT) |  |  |
| 37 |  | formaldehyde/(formaldehyde+acetaldehyde+acetone) | C1/(C1+C2+C3) |  |  |
| 38 |  | acetaldehyde/(formaldehyde+acetaldehyde+acetone) | C2/(C1+C2+C3) |  |  |
| 39 |  | acetone/(formaldehyde+acetaldehyde+acetone) | C3/(C1+C2+C3) |  |  |
| 40 |  | acetone/formaldehyde | C3/C1 ratio |  |  |
| 41 |  | acetone/2-butanone | C3/C4 ket ratio |  |  |
| 42 |  | 2-butanone+butanal+2-pentanone+pentanal | C4+C5 total |  |  |
| 43 |  | (2-butanone+butanal)/(2-pentanone+pentanal) | C4/C5 ratio |  |  |
| 44 |  | 2-butanone/OT | C4H8O/OT |  |  |
| 45 |  | butanal/OT | C4H8O/OT |  |  |
| 46 |  | pentanone /OT | C5H10O /OT |  |  |
| 47 |  | pentanal/OT | C5H10O/OT |  |  |
| 48 |  | hexanal/OT | C6H12O/OT |  |  |
| 49 |  | heptanal/OT | C7H14O/OT |  |  |
| 50 |  | octanal/OT | C8H16O/OT |  |  |
| 51 |  | nonanal/OT | C9H18O/OT |  |  |
| 52 |  | pentenal/OT | C5H8O/OT |  |  |
| 53 |  | hexenal/OT | C6H10O/OT |  |  |
| 54 |  | hydroxy-acetaldehyde/OT | C2H4O2/OT |  |  |
| 55 |  | hydroxy-acetone/OT | C3H6O2/OT |  |  |
| 56 |  | hydroxy-2-butanone/OT | C4H8O2/OT |  |  |


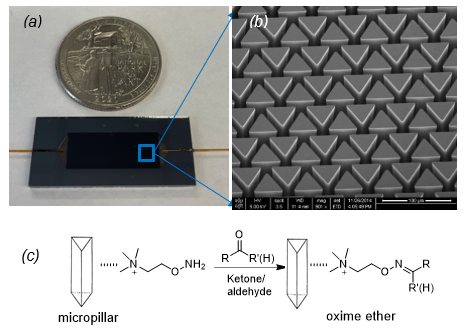


**Figure S1**. (a) Optical micrograph of a fabricated microreactor compared with a dime coin. (b) SEM picture of the micropillar array. (c) Schematic of ATM coated on micropillars and reaction with ketones and aldehydes to form the corresponding oxime ether adducts.


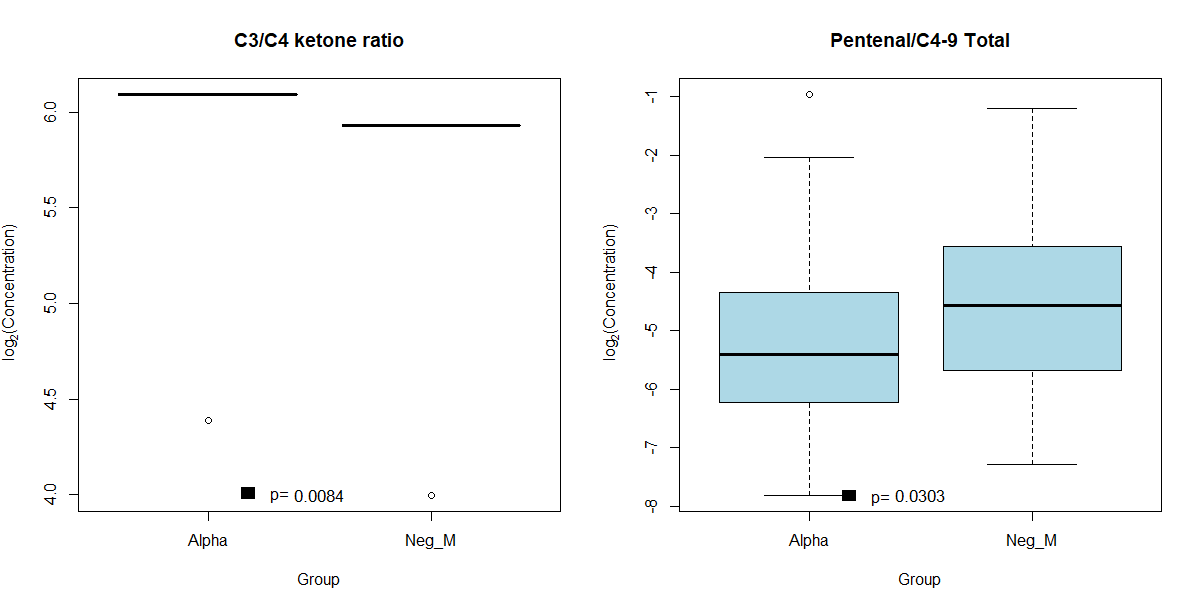


**Figure S2**. Box plots of the biomarkers developed for multivariable model to distinguish COVID-19 Alpha variant positive and negative groups.


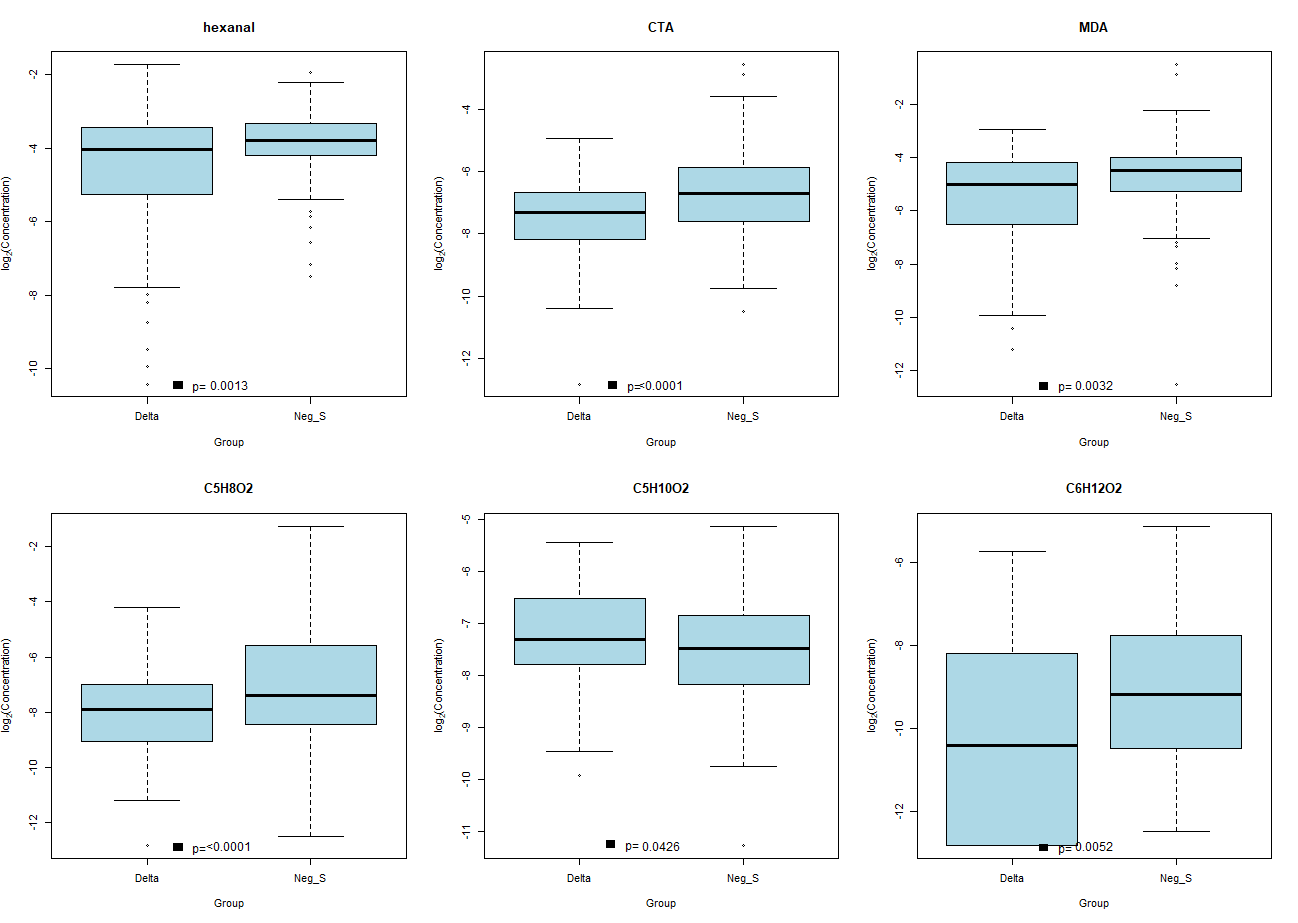

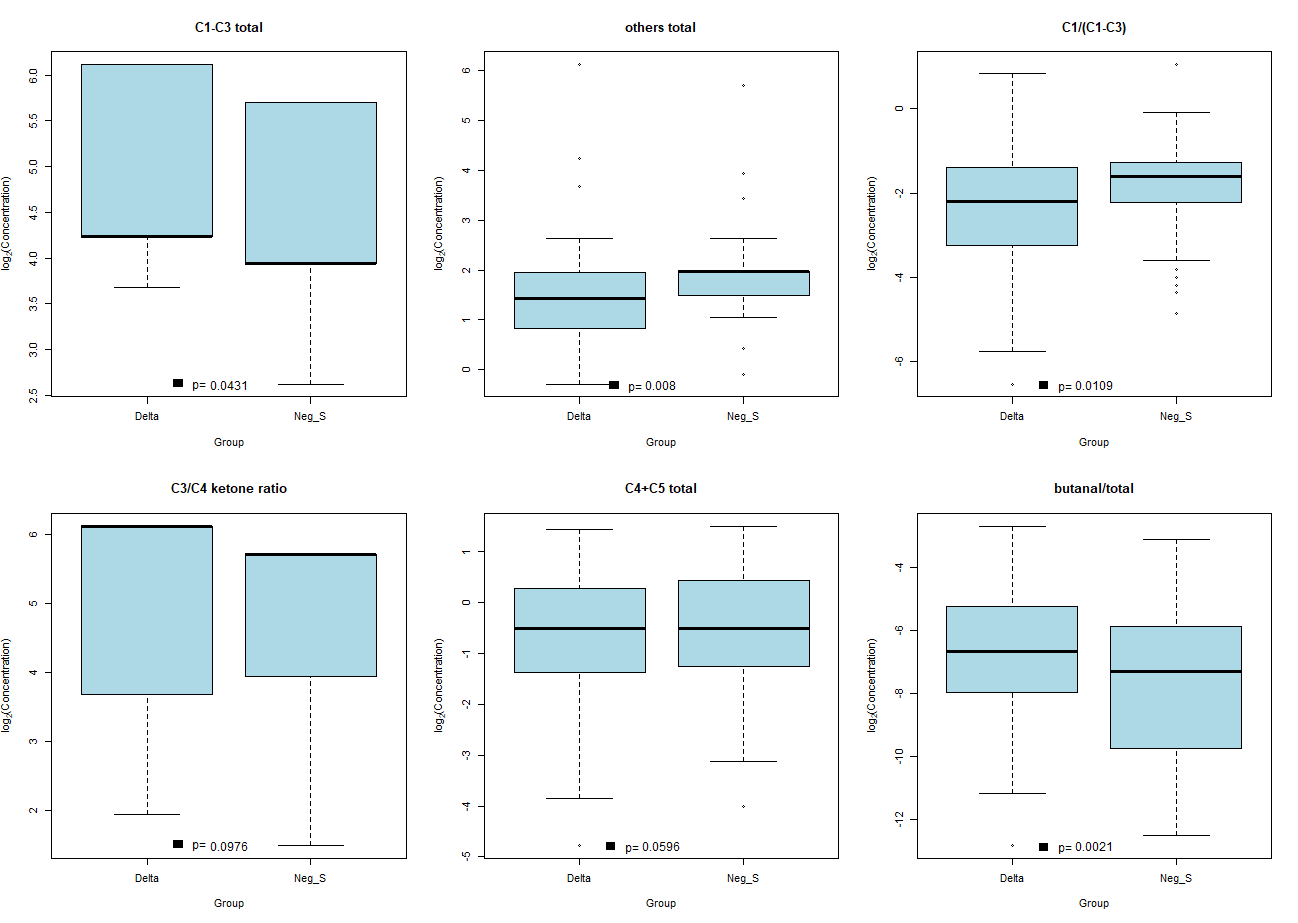


**Figure S3**. Boxplots of the biomarkers developed for the multivariable logistic model to distinguish COVID-19 delta variant positive and negative groups.


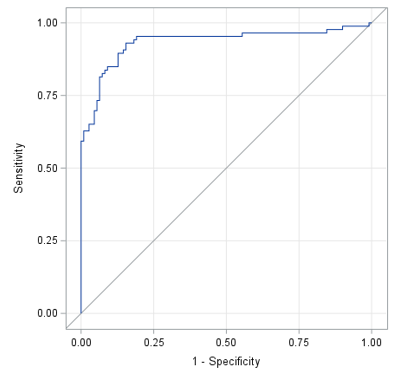


**Figure S4**. ROC curve (AUC = 0.9332) for the multivariable logistic model to distinguish COVID-19 delta variant positive and negative groups with twelve features of hexanal, crotonaldehyde, malondialdehyde, hydroxy-pentenal, hydroxy-pentanal, hydroxy-hexanal, C1+C2+C3, other total (excluded C1, C2, C3), formaldehyde/(C1+C2+C3), acetone/2-butanone, C4+C5, butanal/OT.


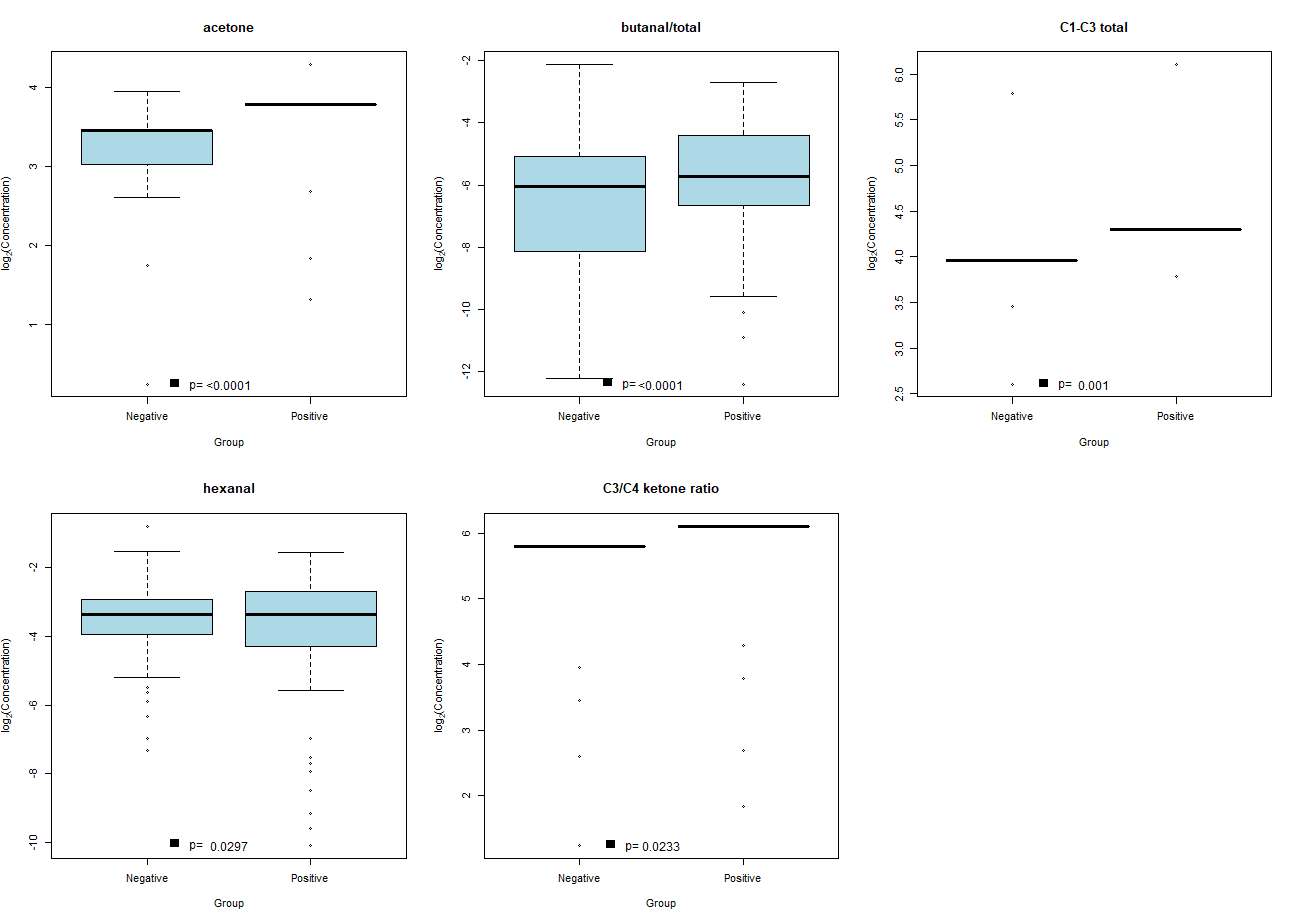


**Figure S5**. Boxplots of the biomarkers developed for the multivariable logistic model to distinguish all COVID-19 positive and negative groups.

**
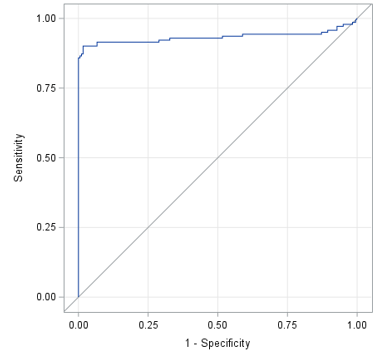
**

**Figure S6**. ROC curve (AUC = 0.9327) for the multivariable logistic model to distinguish all COVID-19 positive and negative groups with features of acetone, hexanal, C1+C2+C3, acetone/2-butanone and butanal/OT.


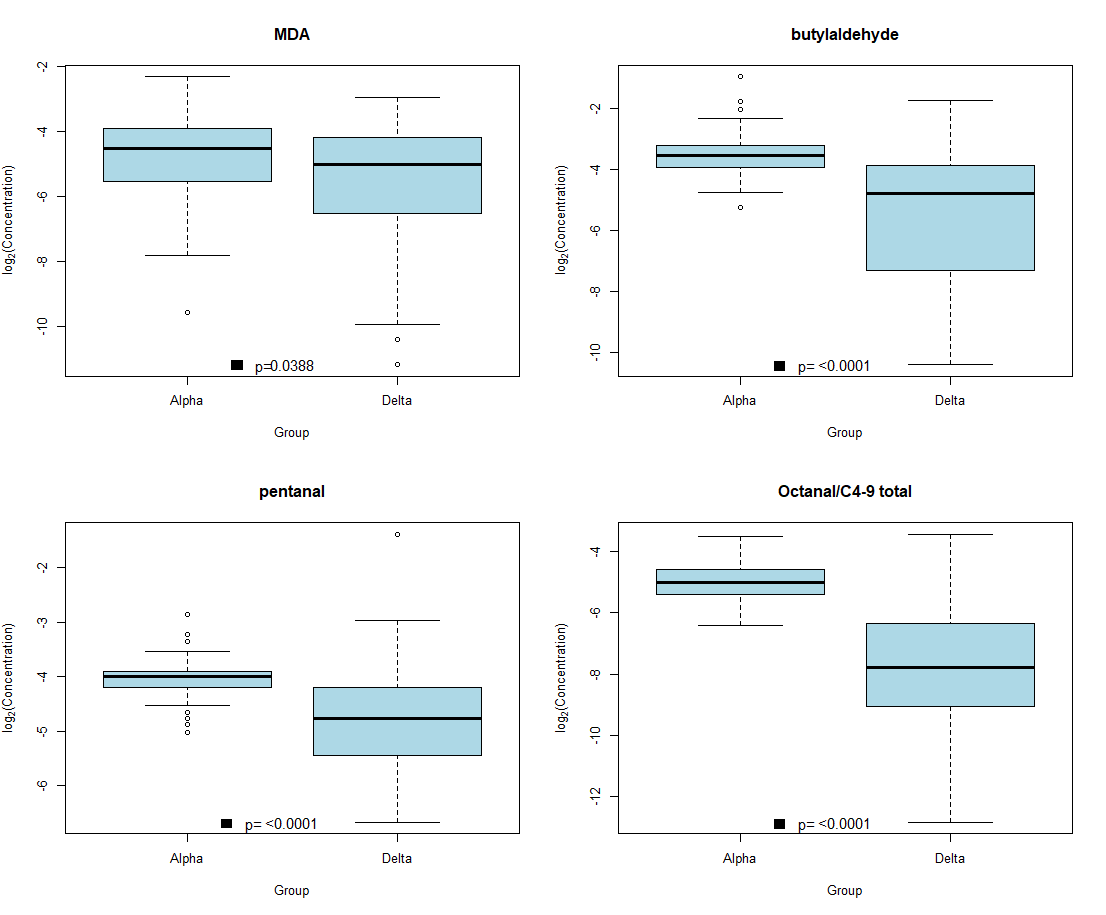


**Figure S7**. Boxplots of the biomarkers developed for the multivariable logistic model to distinguish COVID-19 Alpha wave and Delta wave groups.


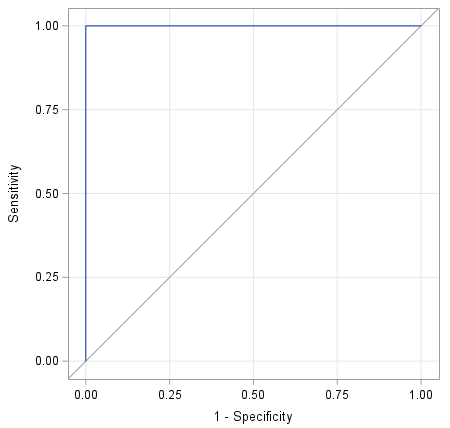


**Figure S8**. ROC curve (AUC = 1.0000) developed for the multivariable logistic model to distinguish COVID-19 Alpha positive and Delta positive groups.


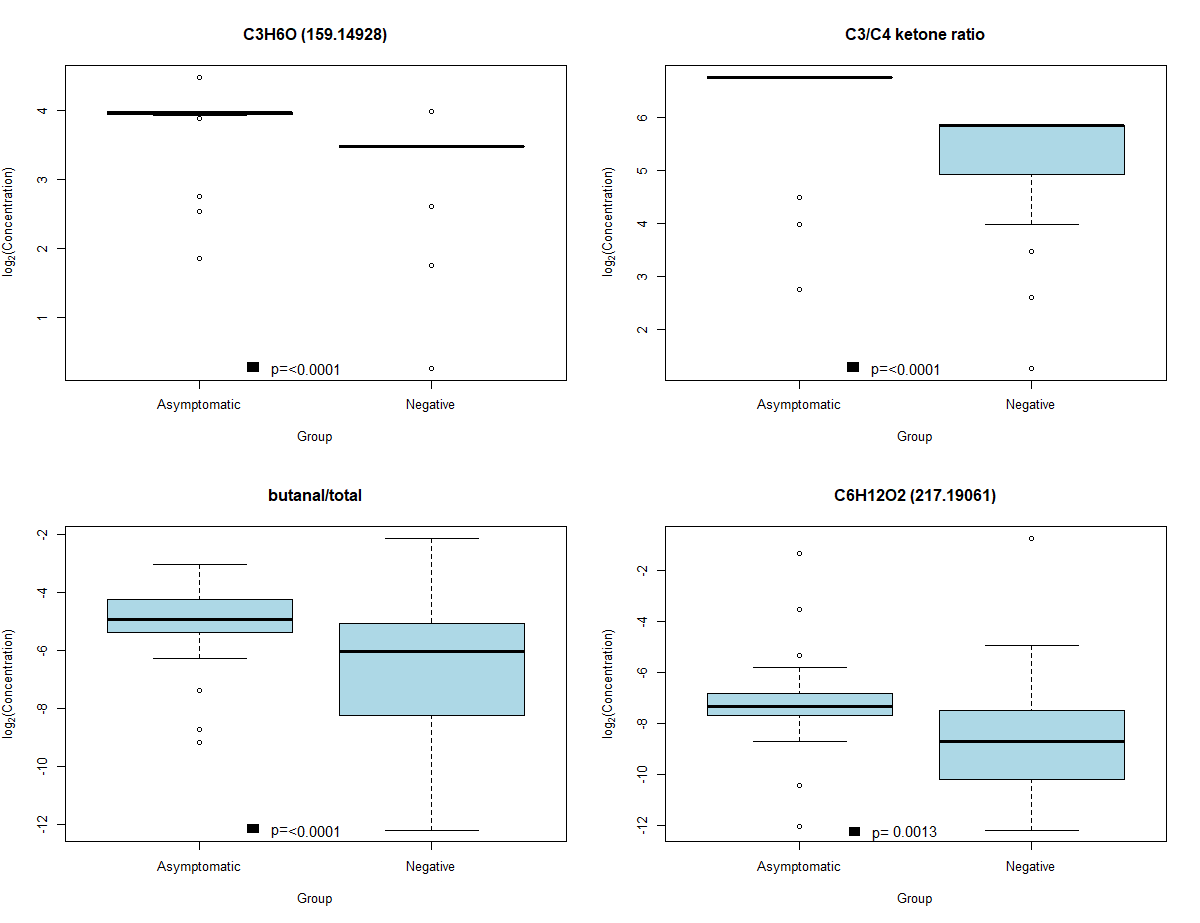


**Figure S9**. Boxplots of the biomarkers developed for the multivariable logistic model to distinguish asymptomatic COVID-19 positive and negative groups.


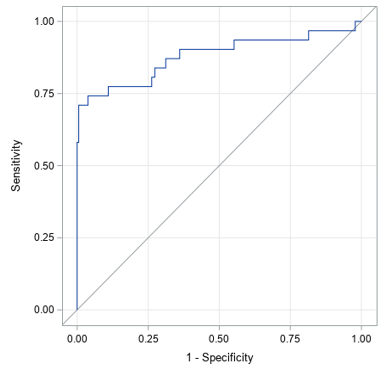


**Figure S10**. ROC curve (AUC = 0.8800) for the multivariable logistic model to distinguish asymptomatic COVID-19 positive and negative groups with features of acetone, hydroxy-hexanal, acetone/2-butanone and butanal/OT.
